# Role of Restorativeness in Improving the Psychological Well-Being of University Students

**DOI:** 10.3389/fpsyg.2021.646329

**Published:** 2021-08-20

**Authors:** Nurul Ain Nabilla Mohd Yusli, Samsilah Roslan, Zeinab Zaremohzzabieh, Zeinab Ghiami, Noorlila Ahmad

**Affiliations:** ^1^Universiti Putra Malaysia, Serdang, Malaysia; ^2^Institut für Biochemie, Deutschen Sporthochschule Köln, Cologne, Germany; ^3^International Islamic University Malaysia, Kuala Lumpur, Malaysia

**Keywords:** attention restoration theory, psychological well-being, restorativeness, university students, environment

## Abstract

Many university students experience high levels of study-related fatigue, hence, necessitating opportunities for restoration. They could potentially benefit from campus-based physical activities that provide them with effective restoration breaks and allow them to return to their studies cognitively refreshed. Thus, a cross-sectional study was conducted to assess the association between perceived restorativeness among postgraduates and their psychological well-being by using the four constructs of Kaplan's attention restoration theory (ART): fascination, being away, extent, and compatibility. In this study, nature view windows were also used as a moderator. Malaysian postgraduate students [*n* = 192; 94 females; age in years (*M* = 30.64, SD = 2.73)] completed the Ryff's scale of psychological wellbeing (PWB) and perceived restorativeness scale for activity (PRAS). This study used the partial least squares-structural equation model (PLS-SEM) to examine these relationships. The results demonstrate that three ART constructs, namely, being away, fascination, and compatibility, are significant predictors of psychological well-being across the sample size. Furthermore, for participants who reside in university dormitories, windows that overlook nature can enhance the relationship of being away, compatibility, and fascination to psychological well-being, compared with those with less natural views. Thus, this study confirmed the moderating effect of nature view windows and provided insight into the ART constructs that facilitate and enhance restorative experiences. By strengthening ART with additional factors, this study has also contributed toward the improvement of the psychological well-being of university students.

## Introduction

One of the major concerns among administrators at institutions of higher learning is the psychological well-being of their students. In their journey toward attaining a university degree, it is common for students to encounter and experience many firsts, some of which can be demanding. Most of these situations require students to tap into their directed attention capacity, an effort that could be extended. Thus, students spend a lot of time learning, studying various courses, solving problems, completing assignments, collaborating on projects, preparing presentations, sitting for tests, and partaking in extracurricular activities, all of which require a direct state of attention (Fu and Cheng, 2017; DiPlacito-DeRango, 2021). As these situations are cognitively demanding, it is evident that they will highly tax the capacity of students to stay focused, thus leading to attention fatigue. In as early as 1995, Tennessen and Cimprich (1995) shone the spotlight on the vulnerability of the university student population to heightened fatigue risk for directed attention. A diminishing capacity to direct attention may impede the academic success of students. It is not uncommon for university students to report that their fatigue of directed attention undermined their ability to function. Consequently, this fatigue impacts their concentration ability, tolerance to irritability, and task accuracy (Hodson and Sander, 2017). Furthermore, during these occurrences, there are few opportunities for breaks that will allow these students to reclaim sufficient relaxation and restoration of directed attention.

Under these conditions, where students are exhausting their cognitive resources, fatigue is likely to be accelerated and a declining psychological balance becomes noticeable. Lazarus (1966) defines stress as “demands made by the internal or external environment that upset balance, thus influencing physical and psychological well-being and requiring actions to restore balance” (p. 19). Correspondingly, most university campuses incorporate and embed restorative spaces into their larger learning spaces. Students can play, rest, and restore in recreational spaces such as open areas, green spaces, and wellness centers. Most universities also boast sports facilities and extracurricular clubs for students to engage in play. However, the differences in these environments could result in differing restoration possibilities, which, in turn, lead to different psychological impacts and restorative qualities. Additionally, past studies have found that university students who often engage in physical activities in green spaces tend to report many positive health impacts both mentally and physically (e.g., Holt et al., 2019).

Most university students are already engaged in activities deemed relaxing and even restorative. However, it is important to understand how they can adjust the way they use their campus resources to establish the restorative process. The first key area of understanding is the characteristics of a fully restorative experience so that it can be incorporated into the daily routines of students. In as early as 1995, Kaplan (1995) developed the attention restoration theory (ART) to explain the restorative experience of an individual through engagement in the abovementioned activities. Attention restoration theory posits that, when the mental effort is exerted, an individual will experience directed attention fatigue. Therefore, an ideal environment for restoration and recovery should exhibit four characteristics, namely, being away, fascination, extent, and compatibility (Kaplan, 1995).

Apart from that, another benefit of the campus setting includes the provision of effective restoration during breaks in dorm rooms; students can resume their work with a refreshed and restored cognition just by, for example, enjoying the natural views outside their windows (Sandifer et al., 2015). According to Kaplan (1995), ART is a micro-restorative experience. In this state, not only is frustration contained but the well-being of students is also promoted (Benfield et al., 2015), resulting in the achievement of optimum levels of psychological functioning and experience (Anglim et al., 2020). Furthermore, exposure to green views through windows has been associated with improved well-being and lower physiological enthusiasm and anxiety among students (van den Bogerd et al., 2020). Past research has also shown that, when nature is viewed through windows, an individual can effectively restore attention (Mitchell and Popham, 2008). Similarly, an early study observed that, when college students enjoy natural views through their dormitory windows, they are able to score higher in tests that demanded directed attention, as opposed to students who only had partial natural views or windowless dormitories (Tennessen and Cimprich, 1995). In this context, window views act as a stress prevention tool and provide opportunities for restoration.

Far too often, university administrators presume that the sole function of a well-tended, landscaped campus is to draw and retain student enrollment (Hajrasouliha and Ewing, 2016). Hence, students seldom get to enjoy restorative experiences, let alone attain optimum levels of restoration. This also reduces their cognitive performance and adversely impacts their physiological well-being. This observation underscores the importance of examining the distinct aspects of ART because current literature has limited studies on the potential of nature for restorativeness. This problem is compounded by the fact that even a small number of published studies have reported conflicting results. This impedes the understanding needed to observe the current and actual scope of the restorative processes residing in the psychological well-being of university students. Inherently, as different individuals prefer different environments, because we cannot assume everyone enjoys nature, further investigation is needed on the ART constructs that can influence the psychological well-being of university students in order for their campuses to provide tailored restorative experiences at the individual level. In addition, most past studies on campus activities, restorativeness, and well-being were mainly conducted in Western countries, while those conducted in the Southeast Asian context, like Malaysia, remain rare. According to Muslim (2017), the nature-acculturation process begins at the onset of the experience of a child in green spaces, which implicates his/her preferences and behaviors toward nature-based activities and all environmental aspects of life. In tropical countries like Malaysia, researchers have found that Malaysian youths, compared to other groups, tend to profess a psychological bond with nature, as they view humanity as a composite of nature, which is a perspective that developed in the early stages of their lives (Muslim, 2017). Thus, it can be argued that campus activities and views of nature from dorm windows play an important role in the lives and health of Malaysian university students.

Against this backdrop, this study is the first to examine ART with its variable representation of being away, extent, fascination, and compatibility as a relationship between the perceived restorativeness of students based on their preferred activities and levels of psychological well-being. The second objective of this study is to explore whether nature window views moderate the relationship between ART constructs and the psychological well-being of university dormitory students who have more natural views from their windows compared to those who do not.

## Literature Review

### Psychological Well-Being of Students

The composition of psychological well-being includes positive relationships with others, personal mastery, autonomy, a sense of having a purposeful and meaningful life, and personal growth and development (Ryff, 2018). This six-factor composition influences the overall psychological state of students when they are on their university campuses. Many studies have reported the prevalence of concerning psychological and mental disorders at universities around the world (Holm-Hadulla and Koutsoukou-Argyraki, 2015). Similarly, several studies have found that Malaysian university students have experienced a decline in psychological well-being (Roslan et al., 2017). Students have to deal with numerous demanding campus conditions that can cause and/or elevate student stress in both the short- and long-term: (1) being on university campus throughout their course duration (4 years or more); (2) being far from home; (3) engaging in intentional, structured, and unstructured learning environments; (4) navigating work-related encounters. In addition, Pedrelli et al. (2015) have observed non-academic stressors that can impact university students, such as added responsibilities in personal money management and home management and carrying out obligations as a roommate. Furthermore, a Universiti Putra Malaysia (UPM) study on a cohort of medical students reported the prevalence rates of stress at 16.9%, depression at 24.4%, and anxiety at 52% (Fuad et al., 2015). Gallego et al. (2014) also raised the alarm on academic stress heightening mental anxiety, which, in turn, impacts the attention processes of students and resulting in potential adversities in their learning and academic performances. Inevitably, the cohort of university students is more vulnerable to attention fatigue.

For new students, they have to deal with new situations in an unfamiliar university setting, all of which demand their directed attention. Essentially a finite resource, Kaplan (2001) states that directed attention is employed for task completion that requires focus, which is an effort to block off unimportant environmental distractions. An example of an unfamiliar setting that students must adjust to is their residential quarters. Living in a dormitory means settling in with modified behaviors in compliance with new rules and constraints. This runs simultaneously with the requirements to meet a new and higher level of academic expectations and demands (Tennessen and Cimprich, 1995). When a student is able to overcome these campus-related sequential changes and adaptations, as described by White et al. (2010), only then would he/she then consider the academic year as successful. Past studies have shown that, post-examination, university students have reported declining levels of directed attention in a baseline comparison (Kulsoom and Afsar, 2015).

However, the development of attentional fatigue is salient in the university experience; perceived campus culture plays a role in how students may seek assistance. Hipp et al. (2016) argued that, for fatigued students, a surrounding natural environment rich in affordances might initiate and sustain restorative experiences that are critical for daily functioning, especially if students perceive that their campus has restorative potential.

### Restorative Environments

Restorative environments are defined as spatial resources that enable recovery from stress and recalibration of personal adaptive resources in meeting everyday demands, specifically by enhancing concentration (Frumkin et al., 2017). In a restorative environment, the directed attention of an individual, which is a pertinent learning resource, is sustained and restored, particularly when it has been exhausted by previous stressful situations (Lin et al., 2019). Researchers have established the usefulness of campus environments for benefits such as stress restoration, attention fatigue reduction, and the enhancement of cognitive functions. The wide variety of natural spaces incorporated into campus grounds (Liprini and Coetzee, 2017) also provide unlimited and cumulative opportunities (Ekkel and de Vries, 2017) for restoration. The location of these green spaces may vary in distance, ranging from an annex or a walking distance from a building, open areas near the center of a campus, to peripheral footpaths. In addition, a university campus usually incorporates restorative spaces into its larger learning spaces, which provide ample opportunities for play, rest, and restoration. These restorative spaces include open and green spaces, sports recreational facilities, societies and clubs, and even wellness centers. As these settings are distinct, they offer different levels of restorative qualities. Therefore, based on these observations, we expect different kinds and degrees of impact on the wellbeing of students, namely, psychologically.

As mentioned previously, earlier studies have established campus experience as the most useful restorer against stress. It also reduces attention fatigue and enhances the ability of an individual to function cognitively (e.g., Williams et al., 2018). Given this, university administrators can encourage students to exploit their university years on campus positively by engaging in physical activities. Researchers have found that the relationship between physical activity and stress reduction is a pathway from nature to wellbeing in the presence of emotional and attentional interaction (Hartig et al., 2014). In particular, Heywood (1978) reviewed the literature on physical activities for leisure and suggested that activities that are recreational and physical in nature can restore attention because participants tend to disengage from attention fatigue factors through such activities, thus leading to reduced tension, feeling energized, and a boost in mental wellness. In addition, physical activities done leisurely have been described as a coping mechanism for dealing with daily stressors (Iwasaki et al., 2006). Therefore, campus spaces potentially serve as sanctuaries for stress relief and restoration by hosting recurrent physical activities (Markevych et al., 2017).

### Attention Restoration Theory

In this study, the researchers framed restorative environments within ART, as this theory explains the restoration experiences of an individual through physical activities (Kaplan and Kaplan, 1989). As mentioned, many positive activities qualify as relaxing and, thus, possibly restore directed attention. ART posit that a restorative environment must offer certain qualities in order to provide the needed restorative experiences. Furthermore, the principles of ART explain why university students may achieve restoration in post-mental fatigue. Even though this theory explains the individual–environment interactions toward attention restoration, it may also offer a broader explanation of environmental aspects where certain activities may be considered vital restorative sources. Therefore, we sought to investigate the relationship between perceived restorativeness during activities and psychological well-being among university students. As per ART, this study focused on cognitive aspects and observed activity engagements in university green spaces that can restore direct attention (Kaplan, 1995). Specifically, we examined the four suggested necessary properties of a restorative environment according to ART, namely, being away, fascination, extent, and compatibility.

In terms of psychology, being away relates to the act of escaping by cognitively engaging in activities in an environment that contrasts everyday settings. To achieve a sense of being away, one does not need to distance him/herself from the environment physically. Scopelliti and Giuliani (2004) suggested that restoration can be attained conceptually rather than through a physical change. The Psychological Recovery Theory (PRT) explains that being away is comparable to being psychologically detached when the individual undergoes a “psychological experience of mentally disengaging from demands during free time, and it involves distraction from task-related thoughts” (Ragsdale et al., 2011, p. 159). Aside from that, resource depletion can be prevented when an individual engages in activities that differ starkly from daily routine or regular demands. While being in a new or novel environment is not required to experience “being away,” the individual still needs to distance him/herself from attention-depleting environments in a physical or mental sense. The fundamental point to establish in the thoughts of an individual is that he/she has claimed a certain distance from stressors and everyday obligations; thus facilitating restoration.

Extent refers to the magnitude of a location in terms of satisfactorily offering opportunities for being away for restoration. It concerns the connectedness and the scope that the individual feels for a location. Connectedness occurs when characteristics or features interweave and interconnect, forming coherence. Scope, on the other hand, refers to the perception of an excellent imaginary experience that has yet to be embarked upon (Wendelboe-Nelson et al., 2019). Combining the two concepts describes the terminology coined by researchers of “a whole other world.” Interestingly, Kaplan (2001) explained that environmental extent can be conceptualized by using cognitive maps, which are mental structures that are constructed from experienced ideation of concepts or objects in the past. Therefore, upon encountering unfamiliar settings or situations that could pose as stressors, creating another cognitive map could allow an individual to tap the restorative properties in an environment.

The fascination dimension was later refined as “soft fascination” in a book by Kaplan and Kaplan (1989, p. 192). Fascination refers to the stimulus for which passive attention is sufficient yet integral to restorative experiences. It must be noted that this stimulus must be interesting enough to lock in a certain degree of attention, albeit an involuntary one. The best example of a fascinating stimulus is watching a sunset in a natural setting (Norling et al., 2008). Another significant point on this property is that fascination is built upon connectedness and scope. Although humans are driven to recognize stimuli even if the recognition of it is difficult or uncertain, events are hard to predict; thus, the state of fascination remains unsustainable if the stimulus is disconnected from a broader framework. In particular, fascination allows us to recover our depleted cognitive resources.

Compatibility refers to the inclination-environment fit of an individual, where the engagement with an environment is defined as the “function of personal intentions, as well as environmental dictates” (Hartig et al., 1997, p. 5). Thus, the focus of this property is placed on the extent of support that a particular environment can provide as a host to the desired activities of individuals. Kaplan and Talbot (1983) suggested that, by using the principle of compatibility, it might at least be possible to begin to define what constitutes a supportive and restorative setting. Since helping people too easily can disrupt adaptive functioning, a creative environment encourages people to build and depend on their coping skills to ultimately affect their well-being.

To date, several studies have investigated the application of ART in clinical and non-clinical populations, with these studies varying with the type of “nature” intervention (e.g., viewing pictures vs. being immersed in an environment), cognitive task and mood measure used, and whether the manipulations were between- or within-subjects (e.g., Earle, 2017). However, there is limited research on the application of ART to psychological well-being. Therefore, the first aim of this study is to investigate the effects of the ART components on psychological well-being among Malaysian university students.

### Nature Window View

Since many campus activities cause the onset of mental fatigue among university students, campus settings ought to support effective restoration breaks that arm the students with rejuvenated cognition. Although many university campuses offer an abundance of lush greenery to provide direct access to and views of nature, students tend to take these facilities for granted and, thus, immerse themselves less in green spaces than they should (White et al., 2019). Consequently, Benfield et al. (2015) and Felsten (2009) have suggested a view of nature through the window as a method for restoring attention to assess how students perceive the restorative values of the indoor campus environment. As mentioned, ART predominantly focuses on explaining how human cognition responds to a specific environment (Kaplan, 2001).

The broad concept of ART, therefore, brought forth an imminent extension. Norling et al. (2008) made headway when they incorporated behaviors into the original ART model. This was informed by past healthcare studies that showed that access to window views can impact the health outcomes of individuals (Devlin and Arneill, 2003). Furthermore, Ulrich (1983) reported that patients recuperating in rooms with nature view windows enjoyed prompt post-operative care and depended less on painkillers compared to patients in windowless rooms. Subsequently, the apartment residents in a study by Kaplan (2001) professed that their greater life satisfaction and sense of well-being were contributed to by the fact that they were afforded views of natural elements from the windows of their units. Recently, researchers reported the positive connection between viewing nature through windows and attention restoration (Jo et al., 2019).

Similar results were reported in research on built environments. Taylor et al. (2002) reported that their sample of urban girls displayed higher levels of self-discipline, concentration, impulse inhibition, and the ability to delay gratification because they had visual access to green spaces from their homes. By pairing the abovementioned theoretical approach on cognition-restorativeness and the architectural trends promoting the quality of outdoor educational settings, this study examined the effects of access to natural green visuals in a university classroom setting. Meanwhile, continued research interest was shown in the effects of biophilic and natural environments in the context of daily living, often in the availability of or accessibility to nature visuals *via* windows (Bolten and Barbiero, 2020). Corraliza et al. (2012) also highlighted nature as a moderator of stress in urban children. While there is some evidence to support the idea that windows with natural views can generate lower levels of stress, very little is known about the moderating effects of natural window views on the relationship between ART constructs and the psychological well-being of university students.

### Aims and Hypotheses

**Figure 1:**
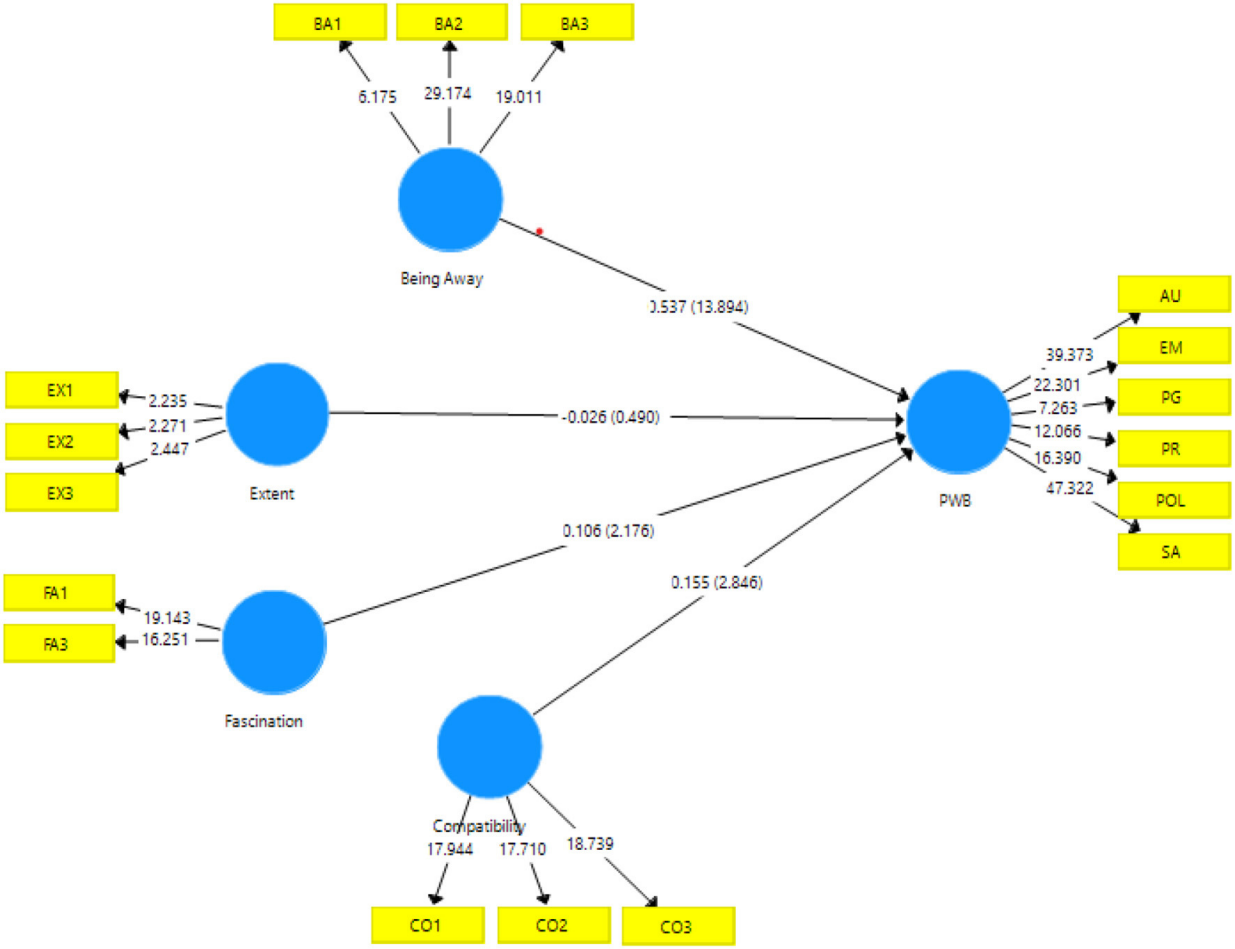
The results of standardized regression weights.

The first goal of this study was to examine the relationship between perceived restorativeness during campus activities and psychological well-being among university students using the four ART constructs. Based on ART theories and previous studies (Trigwell et al., 2014; Olivos and Clayton, 2016; Viviers, 2016; Neilson et al., 2020), the following four hypotheses for the first aim of this study were tested (see Figure 1):

H_1_: There is a positive relationship between being away and psychological well-being.
H_2_: There is a positive relationship between the extent of restorative activities and psychological well-being.
H_3_: There is a positive relationship between fascination and psychological well-being.
H_4_: There is a positive relationship between compatibility and psychological well-being.

In addition, many researchers have shown that gazing at a natural view from a window in the living environment of an individual may be an easily accessible “micro-restorative” activity (Tennessen and Cimprich, 1995; Collado, 2017; Korpela et al., 2017; Engell et al., 2020). Moving forward, the second aim of this study was to investigate the moderating role of a nature window view in relation to ART constructs and psychological well-being. It has been assumed that students who have more natural views from their dormitory windows would show higher levels of psychological well-being than those who have less natural window views; therefore, we formulated the following hypotheses:

H_5_: Nature window views moderate the relationship between being away and psychological well-being.
H_6_: Nature window views moderate the relationship between the extent and psychological well-being.
H_7_: Nature window views moderate the relationship between fascination and psychological well-being.
H_8_: Nature window views moderate the relationship between compatibility and psychological well-being.

## Materials and Methods

### Participants and Procedures

A cross-sectional survey design was employed in this study. The participants were aged 18 years and above and were students of Universiti Putra Malaysia. To fulfill the criteria to employ PLS-SEM, this study required 210 sample sizes, as the sample size to estimate the parameters ratio must be at least 10:1 (Hair et al., 2011). A stratified random sampling technique was utilized to gather the participants. Stratified random sampling is a type of probability sampling that requires the division of a population into smaller groups, which are called strata.

Within the Faculty of Educational Studies (with six departments) of the aforementioned university, we focused on and selected students who were enrolled in the master's degree programs (in classroom settings). The main inclusion criteria are as follows: (1) the Malaysian master's students have completed an exercise class and participated in at least one type of physical activity on the university campus such as jogging, fast walking, badminton, or running; (2) the master's students have lived in the dormitories and their windows had one of these two views: natural views of the lake, bushes, and trees or views of all built structures such as buildings, streets, or parking lots. In this study, the participants were also stratified by window view types, so there were approximately equal sizes in each group. Of the 210 participants, 105 were selected from university dorms with barren window views and 105 were selected from university dorms with nature window views. Although 210 participants were initially enrolled, the valid sample was reduced to 192 participants after incomplete questionnaires were removed.

### Instruments

The questionnaire consisted of three parts: Part A had four items to assess the demographic variables of the respondents; Part B provided a 12-item perceived restorativeness scale for activity (PRAS) (Norling et al., 2008); Part C contained Ryff's (2018) 42-item psychological well-being (PWB) scale-revised.

In environmental psychology studies, experienced restoration measures are usually obtained using the perceived restorativeness scale (PRS; Negrín et al., 2017), the restoration outcome scale (ROS-S; Subiza-Pérez et al., 2017), and the PRAS (Norling et al., 2008). While the PRS is used to assess the restorative potential of environments, the ROS measures positive affect and reflection, which represent the possible restoration outcomes. In our study, the PRAS was used to assess the restorative potential of physical activity among university students. The PRAS is relevant to restorative activities because it offers a theoretical basis for understanding the psychological perceptions that individuals bring to the experience of an activity. The PRAS lists specific activities that reflect all four restorative qualities of an environment. The 12-item PRAS measures perceived restorativeness dimensions present in nature activities; specifically the being away, extent, fascination, and compatibility that are offered by these activities. Being away was measured by three items, with the sample item: “Participating in physical activity helps me get away from it all.” Next, coherence or extent was measured by three items, with the sample item: “For me, physical activity has qualities that draw me further in.” Fascination was measured by three items, with the sample item: “For me, physical activity has many fascinating qualities.” The last three items measured compatibility, with the sample item: “Participation in physical activity helps me achieve my activity goals.” A 7-point Likert scale measured all the items, ranging from 0 (not at all) to 6 (completely). The Cronbach's alpha coefficient for this scale was 0.949.

The PWB Scale (Ryff, 2018) section contained in the questionnaire used by this study contained the following six subscales: self-acceptance (sample item: “When I look at the story of my life, I am pleased with how things have turned out”), positive relationships with others (sample item: “Most people see me as loving and affectionate”), autonomy (sample item: “I am not afraid to voice my opinions, even when they are in opposition to the opinions of most people”), environmental mastery (sample item: “In general, I feel I am in charge of the situation in which I live”), purpose in life (sample item: “I have a sense of direction and purpose in life”), and a sense of personal growth (sample item: “I think it is important to have new experiences that challenge how you think about yourself and the world”). Each 7-item subscale was on a 6-point Likert scale. The response consistency was ensured through the provision of positive and negative statements from which the participants could select their answers, specifically from 1 (strongly disagree) to 6 (strongly agree). Here, we reverse-coded the negative items into positive ones: high scores indicate high psychological well-being and vice versa. For this scale, the Cronbach's alpha coefficient scored 0.96.

The type of view from the window was measured by asking, “Is there a view of nature from the dorm room windows?”. The response categories are either “no” (1) or “yes” (2).

### Data Analysis

The researchers used the PLS-SEM to validate the research model developed for this study (Wong, 2013). The authors used the Smart-PLS 3.0 software for data analysis (Bido et al., 2014) and ran the PLS algorithms with a bootstrapping set to 5,000 subsamples (Hair et al., 2011). The PLS method was preferred over other regression models as it could serve the complex study model and the small sample size (*n* = 192); thus proving the suitability of PLS as an analysis technique for this research (Carrión et al., 2016). To analyze the first objective of the study, we employed the SmartPLS (Version 3.2) to examine the structural model. The SmartPLS MGA procedure was applied to analyze the nature window view as a moderator. The model fit was evaluated using the following fit indices to assess goodness-of-fit: standardized root mean residual (SRMR) <0.08, normed fit index (NFI) >0.9, and the exact fit criteria d_ULS_ and d_G_ < the 95% bootstrapped quantile (HI 95% of d_ULS_ and HI 95% of d_G_) (Henseler et al., 2016).

## Results

### Demographic Results

The demographic characteristics of the respondents are displayed in Table 1. There were 192 respondents, with 43.8% ranging from 26 to 30 years old, while 40.1% were aged 25 years old and below. Another 9.4% of respondents were 31 to 35 years old, and 4.1% were 36 to 40 years old. Finally, only 2.6% of respondents were 41 years and older. As for the age distribution, the mean was 30.64 (SD = 2.73). With regard to marital status, 72.4% of the respondents were single. Most participants (63.5%) indicated that their race was of the Malay ethnic group, followed by Indians (35%), Chinese (27%), and other ethnic groups (4.2%). The participants were enrolled in various courses offered by the Faculty of Educational Studies, namely, Educational Technology, Educational Psychology, Physical Education, Sports Science, Technological and Vocational Education, Guidance and Counseling, Educational Administration, TESL, and Curriculum and Instruction. Finally, 39.1% of them preferred to engage in outdoor activities, 21.4% preferred to observe nature, and 39.6% preferred to participate in social activities.

**Table 1:** Demographic characteristics of the respondents.

**Variable**	**Frequency**	**Percentage**
**Gender**
Female	30	15.6
Male	162	84.4
**Age**
18–25	77	40.1
26–30	84	43.8
31–35	18	9.4
36–40	8	4.2
41–50	5	2.6
**Ethnicity**
Malay	122	63.5
Chinese	27	14.1
Indian	35	18.2
Others	8	4.2
**Financial support**
Self-finance	111	57.8
Sponsored	81	42.2
**Marital status**
Single	139	72.4
Married	53	27.6
**Field of study**
Curriculum and instruction	10	5.2
Educational administration	25	13
Educational psychology	37	19.3
Educational technology	18	9.4
Guidance and counseling	12	6.3
Physical education	1	0.5
Sports science	17	8.9
TESL	42	21.9
The teaching of Malay as a first language	8	4.2
Technical and vocational education	22	11.5
**Year of study**
1	54	29.7
2	44	22.9
3	63	32.8
4	20	10.4
5	8	4.2

The descriptive statistics results for the four dimensions revealed that the respondents scored moderate in psychological well-being (*M* = 4.39, SD = 0.61) and perceived restorativeness (*M* = 4.57, SD = 0.76). These results confirmed the ART constructs of being away, fascination, extent, and compatibility using descriptive analysis. The highest score in perceived restorativeness was fascination (*M* = 4.65, SD = 0.85), followed by being away (*M* = 4.62, SD = 0.83), extent (*M* = 4.52, SD = 0.89), and compatibility (*M* = 4.49, SD =.94).

### Measurement Model

According to Hair and Nitzl (2020), convergent validity, reliability, and discriminant validity should be estimated at the measurement model stage. The measurements for convergent validity and reliability are the value of factor loadings, average variance extracted (AVE), composite reliability (CR), and Cronbach's alpha. All items were maintained because the results exhibited factor loadings above 0.6. As presented in Table 2, convergent validity was achieved in each research variable item. According to Hair et al. (2011), acceptable convergent validity is indicated by the values of AVE (0.5) and CR (0.7).

**Table 2:** Partial least squares-confirmatory factor analysis (PLS-CFA) results.

**Construct**	**Loading**	**M**	**SD**	**α**	**rh-A**	**CR**	**AVE**	**VIF**
**BA**		4.62	0.83	0.751	0.790	0.765	0.526	1.185
BA1	0.768							
BA2	0.813							
BA3	0.771							
**CO**		4.49	0.94	0.770	0.773	0.818	0.599	1.309
CO1	0.768							
CO2	0.794							
CO3	0.760							
**EX**		4.52	0.89	0.799	0.746	0.824	0.612	1.060
EX1	0.831							
EX2	0.765							
EX3	0.838							
**FA**		4.65	0.85	0.721	0.724	0.840	0.725	1.193
FA1	0.865							
FA3	0.838							
**PWB**		4.39	0.61	0.824	0.875	0.867	0.526	–
PG	0.528							
POL	0.701							
PR	0.675							
SA	0.840							
EM	0.763							
AU	0.801							

*CR, values of composite reliability; AVE, average variance extracted; VIF, variance inflation factor; M, mean; SD, standard deviation; AU, autonomy; BA, being away; CO, compatibility; EX, extent; FA, fascination; PWB, psychological well-being*.

Using empirical standards, the concept of discriminant validity recognizes degree differences between any two focal constructs. Consequently, this study conducted an analysis of discriminant validity as per the recommendations of Fornell and Larcker (1981) and Henseler et al. (2015); we employed the Fornell–Larcker criterion and the Heterotrait–Monotrait (HTMT) ratio. The Fornell–Larcker criterion provided the square root of the AVE of each of the constructs, which was greater than the correlation values of any other construct. Additionally, in all cases, the HTMT values scored 0.85, which was below the threshold level (see Table 3). Therefore, this study confirmed that the variables of this study could be mutually discriminated in the study.

**Table 3:** Measurement model: discriminant validity.

**Fornell-larcker criterion**						**Heterotrait-monotrait ratio (HTMT)**			
	**1**	**2**	**3**	**4**	**5**	**1**	**2**	**3**	**4**
1. EX	0.782								
2. BA	0.121	0.725				0.177			
3. CO	0.199	0.378	0.774			0.292	0.590		
4.FA	0.191	0.242	0.369	0.851		0.258	0.423	0.576	
5. PWB	0.091	0.618	0.392	0.288	0.725	0.139	0.808	0.464	0.376

*AU, autonomy; BA, being away; CO, compatibility; EX, extent; FA, fascination; PWB, psychological well-being*.

### Structural Model

Upon the validation of the measurement model, path analysis was conducted to test H_1_ and H_4_. The structural model was evaluated by examining the significance of path coefficients, coefficient of determination (*R*^2^), and predictive relevance (Q^2^). The structural model was evaluated using a non-parametric bootstrapping procedure with a resample of 5,000 to generate the β and corresponding *t*-values. This study confirmed that the data fit the model well because the results showed that the SRMR value was 0.03 (<0.08), the NFI was 0.939 (>0.9), and the d_ULS_ < bootstrapped HI 95% of d_ULS_ and d_G_ < bootstrapped HI 95% of d_G_. This study confirmed that the data fit the model well because the results of all the models showed that none exceeded the cut-off value of 0.08 for SRMR values and >0.8 for NFI values (Henseler et al., 2015). To be considered moderate, *R*^2^ should be above 0.33 (Chin, 1998). The results displayed that being away, fascination, and compatibility jointly explained 42.1% (*R*^2^ = 0.421) of the variance in psychological well-being. These results indicated a medium predictive power of the corresponding constructs, which were supported by Q^2^ positive values.

Table 4 presents the path and parameter estimates related to the direct model. The findings showed that the path coefficients of being away (β = 0.27, *t* = 13.894, *p* = 0) were significantly associated with psychological well-being, thus supporting H_1_. Meanwhile, H_3_ was also supported, as fascination (β = 0.106, *t* = 2.176, *p* = 0.03) was significantly related to psychological well-being. Next, H_4_ was supported by the path coefficients that showed compatibility (β = 0.155, *t* = 2.846, *p* = 0.005) being significantly related to psychological well-being, whereas no significance was reported on extent (β = −0.026, *t* = 0.49, *p* = 0.062) toward psychological well-being; thus, the results reject H_2_ (Figure 1).

**Table 4:** Structural model (bootstrapping).

**H**	**Paths**	**Beta**	***t-*value**	***P*-value**	**Bias corrected bootstrap (95%)**	
					**LL**	**UL**
H_1_	BA → PWB	0.537	13.894	0	0.46	0.614
H_2_	EX → PWB	−0.026	0.49	0.0624	−0.179	0.048
H_3_	FA → PWB	0.106	2.176	0.03	0.01	0.193
H_4_	CO → PWB	0.155	2.846	0.005	0.045	0.256

*AU, autonomy; BA, being away; CO, compatibility; EX, extent; FA, fascination; PWB, psychological well-being*.

The moderation effects of nature window views and gender were estimated through a multi-group analysis (MGA), specifically by employing the Henseler-MGA non-parametric technique because it could evaluate the path coefficient differences between the two groups. In the PLS-SEM in this study, this technique also effectively evaluated group differences (Hair et al., 2011). Before conducting the MGA, the study assessed the measurement invariance of the model by following the measurement invariance of the composite models (MICOM) procedure. The results showed that the requirement for the configuration invariance and the compositional invariance of the model were fulfilled, creating the partial measurement invariance needed to conduct the MGA.

In the next phase, the moderating roles of nature window views on the relationships in H_5_ and H_8_ were verified using two different types of window views in the MGA, respectively. The first type refers to the outdoor view, which mainly consists of urban and “concrete jungle” visuals such as buildings or streets. The second type refers to the outdoor view that predominantly consists of nature visuals such as lakes, fields, or parks. As demonstrated in Table 5, significant differences have been observed for the structural relationships postulated in H_5_, H_7_, and H_8_, as the *p*-values of the differences in path coefficients between a nature view from the window and no nature view from the window are all less than *p* < 0.05. Therefore, our results indicate that the nature window view significantly moderates the proposed relationships in H_5_, H_7_, and H_8_. However, the *p*-values of the differences in path coefficients between a nature window view and no nature window view for H_6_ are higher than *p* < 0.05. Therefore, the moderating effect of the nature window view in H_6_ is not supported (*p* = 0.078).

**Table 5:** Hypotheses test by groups: nature window view vs. no nature window view [multi-group analysis (MGA)].

**H**	**Paths**	**Nature window view**		**P(Diff)**
		**YES**	**NO**	
H_5_	BA → PWB	0.17^***^	0.37^***^	0.043
H_6_	EX → PWB	0.13	0.06	0.231
H_7_	FA → PWB	0.53^**^	0.37^**^	0.021
H_8_	CO → PWB	0.29^***^	0.13^***^	0.031

BA, being away; PWB, psychological well-being; CO, compatibility; FA, fascination; EX, extent; The multi-group comparison is based on a non-parametric approach (MGA); ^*^,

^**^, ^***^ indicate significance at the 5, 1, and 0.1% levels.

## Discussion and Implications

Previous studies have proven the restorative effect that nature experiences and outdoor activities can provide for the enhancement of psychological and physiological functioning (Chiang et al., 2017; Elsadek et al., 2019; Browning et al., 2020). This study takes the findings of previous research a step further by investigating the role of restorativeness, specifically on the psychological well-being of university students in Malaysia. To verify this, this study aimed to investigate if the set of environmental qualities in the ART can facilitate the perceived restoration of cognitive resources in students to predict the psychological well-being of Malaysian postgraduate students. This study has also highlighted campus-based physical activities as essential spatial resources that can improve the psychological well-being of students. Moving forward, the current study used four ART constructs to investigate the relationships between the perceived restorativeness of postgraduates and their psychological well-being. To explore these relationships, nature window views were included as a moderator in this study. This shed light on the health benefits of viewing nature through dormitory windows in the university and extended ART to strengthen the psychological well-being of university students.

Having found that being away, fascination, and compatibility were significant factors, this study indicates that spatial activity is necessary on university campuses to benefit university students. This further suggests that the interlocking of the three factors may serve to amplify experiences and emotions on campus. Correspondingly, physical activities on campus are beneficial for university students in terms of reducing stress, remedying mental fatigue, boosting their mood, and restoring mental health. The above findings are consistent with another study that reported a positive association between restorativeness and life satisfaction, while a negative association was found between the stress of university students and burnout (Payne et al., 2020). This study stated that increased restorativeness levels would positively correlate with well-being, herein life satisfaction, and have a negative correlation with distress, specifically stress and burnout. Another important finding of this study was that, when respondents relaxed in a natural environment, their distress levels showed a decrease and their well-being levels increased in comparison to the control group waitlist (after controlling for pre-scores). Additionally, the study of Howell et al. (2011) supports our findings of a positive link between nature and psychological well-being.

The two significant factors that contribute toward restorativeness among postgraduate students are being away and fascination for the first and second hypotheses. Results have shown that getting away from distractions, or in other words, changing a fixated mindset of being stuck in stress and everyday obligations, helps facilitate restoration. These findings suggest that postgraduate students should physically detach from academic spaces and conceptually engage in regular physical activities on university campuses. Subsequently, these students would return to lectures more rejuvenated cognitively after involving themselves in more on-campus activities. These findings support ART, in which the quality of restorative experiences, more precisely explained in terms of fascination and not just the quantity, makes an essential difference in psychological well-being. The relationship between campus activities and psychological well-being is essential because it can extend attention capability toward resilience (Fredrickson, 2001) and general health improvements. Furthermore, this study also found that the moods of postgraduate students were uplifted after experiencing more fascination toward the university campus. This finding is supported by studies that observed and interpreted fascination experience within university environments as leading to greater well-being. However, in contrast, well-being was negated when campus environments were experienced in the absence of fascination. According to Kaplan (1995), the ball is in the individual's court to recognize and seek supportive, conducive environments to support their cognitive restoration efforts. Many studies have promoted the role of campus environments in such a task, even if it may promote or inhibit the belief that healthful choices regarding physical activity are the most accessible way to awaken fascination; thus suggesting that these environments are suitable hosts for reflection and restoration (e.g., Herzog et al., 2003; Horacek et al., 2014).

On the contrary, this study revealed no significant link between extent and psychological well-being. Based on the findings, university students require substantial time to familiarize themselves with their surroundings. Thus, presenting them with new or unusual scenarios may be counterproductive. Tremendous success in evoking extent can be achieved within familiar environments. However, as soon as students are presented with unfamiliar stimuli or experiences, using a cognitive map can be very helpful; it takes away from the restorative properties in the environment. Furthermore, the property of compatibility in H_4_, which is the final quality of an ART environment, was supported in this study. It refers to how university students engage in activities that match and support their desires and inclinations. In seeking compatibility, university students need to stay away from or circumvent unfamiliar places. This requires multiple cognitive maps to be run at once, thus exhausting more energy to familiarize themselves with new environments. Incompatibility with an environment arises in the presence of inappropriate motivation (or extrinsic motivation). This form of motivation reduces the meaningfulness of well-intended activities, thus reducing restorative effects. Toward this end, university students may find that engaging in an entirely new physical activity has no restorative value, especially when they struggle to acquire a novel skill. If the university students engage in an intended physical activity, they would have made adequate preparations. As a result, this study found that compatibility was linked to the psychological well-being of students because they were comfortable with their physical activities on university campuses.

Apart from that, the current study offered insight into the type of campus activities that can be conducted, namely, physical activities, which is a common concern among educational planners. As educators need to support the psychological well-being of their students, the pursuit of academic excellence should be co-driven by mindful activities that provide restorative experiences. It is in the interest of educators to be informed of potentially restorative activities that could be appropriate for their students in order to provide postgraduates with a less intimidating learning environment. Universities can also educate students about the various physiological and psychological benefits of participating in campus-based physical activities such as attention restoration.

The results also indicated that nature window views can moderate the three facets of ART, namely, being away, fascination, and compatibility, and psychological well-being. The findings from the current study suggested that one does not need to view only real nature to gain positive psychological impacts. Instead, seeing nature from the window can also benefit psychological well-being. It was hypothesized that natural views from dormitory windows can improve the psychological well-being of students by increasing their connection with nature. This research also presented preliminary evidence on how watching fascinating scenes can enhance aspects of psychological well-being among university students. This result was confirmed by studies that reported the ways people value simulations of restorative settings for their psychological well-being (e.g., positive emotions, reduced stress, and cognitive fascination; Hartig et al., 2014). According to Kaplan (1995), an individual is responsible for recognizing and seeking supportive and conducive environments to support their cognitive restoration efforts. Many studies have promoted restorative environments as the most accessible way to awaken fascination, suggesting that these environments are suitable hosts for reflection and restoration (Herzog et al., 2003). Additionally, by viewing nature through the windows in the dorm rooms, participants could be frequently reminded of the positive experiences they had in the outdoors, thus leading to increased psychological well-being. Alternatively, seeing nature might have also changed what university students value in their lives. Previous literature has shown that viewing nature scenes decreased the selfish and extrinsic desires of participants (Weinstein et al., 2009). The results of this research validated the work of Raanaas et al. (2012) by proving that those who have natural views from their dormitory windows are better able to direct attention than those with less natural views.

Our results also contributed to the theoretical basis for landscape design beneficial to the psychological health of university students. It is a starting point for further research on the relationship between the characteristics of a visual landscape and the psychological well-being of university students. Our findings helped to reinforce the importance of natural window views in university dorms. The findings here demonstrated that simply putting windows in dorm rooms is not enough; those windows need to overlook nature in order to bring about a positive effect on the stress recovery of students. If dorm rooms have a window view of nature, they can provide more opportunities for contact with nature and help improve stress recovery. Based on this study, it can be concluded that an integrated approach to design is essential to provide a comfortable atmosphere for university students.

## Limitations and Future Research Direction

We realize that this study has limitations. The first limitation is the possibility of a common bias by adopting a single questionnaire to analyze the theoretical complexity of ART. Another limitation is that this study was not designed to conduct any inter-cultural comparison. As a consequence, the results could be constrained in their generalizability. Additional research in different countries would be needed to generalize the findings. The third limitation in this study arises from the validity and reliability of the measures of ART. In validating the measures of directed attention, many authors have found that measures like the PRAS have high validity and reliability. While validation is a useful step in operationalizing the scale, there are some concerns on how well the scale measures directed attention. Within the literature on ART, there is tremendous variability in how directed attention is measured. Systematic reviews (Ohly et al., 2016, Stevenson et al., 2018) of ART literature showed different measures for directed attention in many studies and subsequently highlighting important inconsistencies within this research area. Validation is not a comprehensive evaluation of everything that ART is not; the PRAS scale and factor analysis only support the validity and reliability of the theory but do not tell us about the psychological reality of how nature affects attention. The fourth limitation is that the study is cross-sectional, and all of the conclusions are based on correlations rather than causality. Fifth, when compared to objective measures, self-reports of physical activity are typically exaggerated, which may have resulted in some bias in the studies. The final limitation of this study is the small sample size due to the low numbers of the study population. Therefore, future studies can improve such shortcomings by directly observing the subjects over time. Future studies can take note of these shortcomings when planning future research work by, for example, specifying different measures of ART. Finally, it would be also interesting for future studies to improve a single questionnaire to analyze ART.

## Conclusion

In conclusion, ART is an effective theory that can be employed to investigate the relationship between perceived restorativeness and psychological well-being among Malaysian postgraduates. The results contribute to environmental psychology literature by providing additional empirical support for the capacity of the three constructs of ART (i.e., fascination, being away, and compatibility) in predicting psychological well-being. The results also indicate that nature window views moderate the mentioned ART constructs and psychological well-being. In addition, the results differed according to the nature window view for all predictors, except for the extent of restorative activities. We conclude by noting the importance of enhancing the psychological well-being of postgraduates by providing university dorms with green window views. This study proved that campus-based physical activities amongst Malaysian postgraduates have a significant positive impact on their psychological well-being and mental fatigue. These findings can guide university administrators, landscape architects, policymakers, planners, and designers who are interested in creating more supportive environments for learning.

## Data Availability Statement

The data analyzed in this study is subject to the following licenses/restrictions: permission is needed from the postgraduate student. Requests to access these datasets should be directed to Nurul Ain Nabilla Mohd Yusli, nainnabilla@gmail.com.

## Author Contributions

NM: data collection. SR: supervision and draft. ZZ: data Analysis and review. ZG and NA: visualization. All authors contributed to the article and approved the submitted version.

## Conflict of Interest

The authors declare that the research was conducted in the absence of any commercial or financial relationships that could be construed as a potential conflict of interest.

## Publisher's Note

All claims expressed in this article are solely those of the authors and do not necessarily represent those of their affiliated organizations, or those of the publisher, the editors and the reviewers. Any product that may be evaluated in this article, or claim that may be made by its manufacturer, is not guaranteed or endorsed by the publisher.
